# Extracellular matrix alterations in the skin of patients affected by psoriasis

**DOI:** 10.1186/s12860-021-00395-1

**Published:** 2021-10-29

**Authors:** Mariana Fatima Muaccad Gama Wagner, Thérèse Rachell Theodoro, Carlos D’. Apparecida Santos Machado Filho, Luiza Keiko Matsuka Oyafuso, Maria Aparecida Silva Pinhal

**Affiliations:** 1 Dermatology Department of Centro Universitário Saúde ABC (FMABC), Santo André, São Paulo Brazil; 2 Biochemistry Department of Centro Universitário Saúde ABC (FMABC), Avenida Lauro Gomes 2000, Santo André, São Paulo CEP 09060870 Brazil

**Keywords:** Psoriasis, Heparanase, Heparanase-2, Syndecan-1, Metalloproteinases, TIMP2

## Abstract

**Background:**

Psoriasis is a chronic inflammatory disease dependent upon a complex interaction between genetic predisposition and immunological factors. It is characterized by skin lesions throughout the body, causing great morbidity and affecting life quality. The present study aimed to evaluate the protein and mRNA expression of heparanase-1 (HPSE), heparanase-2 (HPSE2), syndecan-1 (SYND1), metalloproteinases (MMP2, MMP9), and tissue inhibitor metalloproteinases 2 (TIMP2) in skin samples.

**Methods:**

From each psoriasis patient, two samples were collected, one sample from a psoriasis plaque (*n* = 23) and the other sample from non-affected skin (*n* = 23), as well as tissue collected by blepharoplasty from control individuals (*n* = 18). Protein expression was investigated by immunohistochemistry, followed by digital quantification. Quantitative RT-PCR obtained mRNA expression. Statistical analyses were done, and *p* values < 0.05 were considered significant.

**Results:**

A significant increase in protein and mRNA expression was observed in both heparanases (HPSE and HPSE2), and higher protein levels of MMP9 and TIMP2 were observed in the psoriasis plaque compared to the non-affected skin. The data point to a probable activation of MMP2 by TIMP2. Moreover, there was a significant increase in HPSE2, SYND1, MMP9, and TIMP2 in non-affected skin samples from patients with psoriasis than in the control sample (tissue obtained by individuals who do not have psoriasis).

**Conclusions:**

These results show a possible correlation between the characteristic inflammatory process and alterations in the expression of the extracellular matrix in psoriasis. The increased expression of HPSE2, SYND1, MMP9, and TIMP2, even in the absence of psoriatic plaque, indicates that these molecules may be involved with extracellular matrix changes in the initial alterations the psoriatic process and may be candidates for the development of target treatments.

**Supplementary Information:**

The online version contains supplementary material available at 10.1186/s12860-021-00395-1.

## Background

Psoriasis is a chronic inflammatory immune-mediated disease, which affects the skin, joints. It is associated with multiple comorbidities that diminish patients’ quality of life [1, 2]. Frequently, individuals affected by the disease are victims of prejudice that impacts their careers and psychosocial relationships [3–9]. Damevksa and coworkers showed that inflammation induced by psoriasis causes insulin resistance and endothelial dysfunction, which lead to atherosclerosis and, ultimately, to major cardiovascular events. It has been suggested that psoriasis, the early and successful treatment, may prevent the development of such comorbidities [8].

The Executive Board of the World Health Organization (WHO) recognizes psoriasis as a severe non-communicable disease that affects about 3% (125 million) of the world population and 1.3% (5 million) of the Brazilian population [10, 11]. The occurrence of psoriasis correlates with ethnicity and geographic region [12, 13]. The incidence of psoriasis is similar among the sexes. It is estimated that around 45% of psoriasis cases occur before sixteen years of age, and in around 2% of cases, before 2 years of age [14–16].

The Psoriasis Area and Severity Index (PASI) is used to classify mild, moderate, and severe psoriasis, allowing for an estimation of patient treatment efficacy and clinical follow-up [17–21]. Some authors define moderate to severe psoriasis as those with PASI > 10. In contrast, others define moderate psoriasis as PASI between 7 and 12 and severe ones with PASI > 12 [22, 23]. Moreover, there is no specific laboratory marker to classify the severity of psoriasis.

It is known that the extracellular matrix (ECM) is critical during epithelial morphogenesis and modulates cellular signaling since its components can act as ligands for cell surface receptors, co-receptors for growth factors, cytokines, and regulate immune cell dynamics.

The interplay between ECM components such as proteoglycans, metalloproteinases is essential in developing skin diseases. Tissue remodeling processes during psoriasis’ pathogenesis influence immune cell activation and modulate inflammatory response [24, 25].

Previous data show that the heparan sulfate proteoglycan syndecan-1 plays a critical role in modulating psoriasis-like skin inflammation in mice [26]. Syndecan-1 regulates IL-17 production by innate lymphoid cells, a major T cell subset of intraepithelial lymphocytes and natural killer T cells, with important implications for host defense and autoimmune diseases [26].

Heparanase can be double-edged; as a promoter of tumor survival and growth, heparanase can also enhance tumor immune surveillance and tumor cell clearance. Additionally, heparanase has been described to induce the development of psoriasis form of skin inflammation in mice [27–30].

Given such considerations, we decided to investigate whether the proteoglycan syndecan-1 (SYND1), heparanases (HPSE and HPSE2), metalloproteinases (MMP9, MMP2), and TIMP2 might be involved in the alteration of extracellular matrix (ECM) in psoriasis, using comparative analysis between skin samples obtained from patients with psoriasis and tissues of individuals not affected by the disease.

We also decided to investigate whether there is a different expression profile of such molecules according to a different PASI classification. Alterations of the ECM might help better understand psoriasis’ molecular mechanisms and reveal potential target molecules to improve the disease treatment.

## Methods

### Sample/casuistic

The study subjects were invited to participate in the research voluntarily, and only those who signed the Free and Informed Consent Form (FICF) were included. The Ethics Committee approved the project of the ABC Medical School as no. 676.610. The samples were made up of three groups. Two skin biopsies (4 mm punches) were carried out on each patient with psoriasis, one sample from the central region of the psoriasis plaque (*n* = 23, Psoriasis Group) and the other sample obtained from a non-affected area (*n* = 23, Non-affected Group). The control group (*n* = 18) was obtained from healthy individuals who underwent blepharoplasty surgery. Each sample collected was divided into two parts to perform both assays, RNA extraction (RT-PCR analysis) and immunohistochemistry analysis. To select patients with psoriasis, the inclusion criteria used were: 18 years of age or older, diagnosis of active psoriasis, and no prior treatment for psoriasis. The inclusion criteria for individuals in the control group were 18 years of age or older, in good health with no comorbidities. The exclusion criteria for both groups (control and patients) were a current or past history of neoplasia, active infectious disease, diagnosis of another skin disease, apart from psoriasis, under 18 years of age, pregnancy, or breastfeeding. All samples were collected by the Dermatology Surgery Department of the Centro Universitário Saúde ABC (FMABC) and stored in 10% buffered formalin, embedded in paraffin, and sliced into histological sections of 5 μm. In addition, the samples were also stored in RNAlater (Sigma-Aldrich, USA) for total RNA extraction. The samples were submitted to hematoxylin and eosin (H&E) staining, followed by immunolabeling to analyze protein expression with specific antibodies and mRNA expression by RT-PCR. In the present study, psoriasis was classified as mild for those with PASI < 7, moderate for those with PASI between 7 to 12, and severe for those with PASI > 12, following Brazilian guidelines [22, 23, 31].

### Histopathological analysis

The samples were submitted to hematoxylin and eosin staining, followed by immunolabeling to analyze protein expression with specific antibodies. Two dermatologists reviewed the sections of skin biopsies and control tissues stained with H&E. The immunostaining slides were digitized under a 40X objective using an Aperio CS2 (Leica Biosystems) scanner generating digital files. The slides were then quantified using ImageScope™ software (Aperio Technologies). The epidermis (corneum, spinosum, granulosum, and basal layers) and dermis (papillary and reticular) were evaluated based on the histological characteristics of the control tissues (blepharoplasty) and tissues collected from patients affected by psoriasis.

### Immunohistochemistry

The antibodies anti-heparanase-1 (HPA-1 C-20) anti-heparanase-2 (HPA-2 C-17), anti-metalloproteinase-2 MMP-2 (8B4), anti-metalloproteinase-9 MMP-9 (C-20) and anti-inhibitor of metalloproteinase-2 TIMP2 (H-140) were obtained from Santa Cruz (Santa Cruz, CA, USA) and diluted 1:100. The anti- Syndecan-1 (clone CD138 BB4 MCA681) from the Bio-Rad Company Co. (AbD Serotec®, Oxford, UK) was diluted 1:50. Immunolabeling was carried out using the avidin-biotin-peroxidase complex, following the protocol described by the manufacturer, LSAB+System-HRP kit (Dako North America, Inc., CA, USA) and 3,3′-diaminobenzidine as liquid chromogen - DAB + Substrate Chromogen System (Dako North America, Inc., CA, USA). The sections were analyzed under a TS100 Nikon Eclipse® light optical microscope to identify the areas that best represented the immunolabeling. In each case, the immunolabeling of the image was quantified by computer-assisted image analysis [32]. For each case, microphotographs of 640 × 480 pixels were obtained from consecutive and non-coinciding fields, with a magnification of 400X through an optical microscope using a Nikon Coolpix 4300® digital camera. The images analyzed by the ImageLab® software (Softium Informática®, São Paulo, Brazil) were adjusted to a micrometric scale (μm).

### RT-qPCR (real-time PCR)

Messenger RNA expression of the heparanase isoforms (HPSE and HPSE2) and SYND1 was obtained using real-time RT-PCR. Total RNA was extracted from the samples using the reagent TRIzol® (Ambion by Life Technologies™, California, USA), following the manufacturer’s instructions. Reverse transcription was carried out using the reverse transcriptase enzyme ImPromII™ (Promega Co., Wisconsin, USA) following instructions from the manufacturer for obtaining complementary DNA (cDNA). Quantitative RT-PCR was carried out using specific sense and antisense primers. The mRNA expression was determined by geometric mean using as control the expression of the endogenous ribosomal protein 60S L13A (RPL13a), sense primer 5’TTGAGGACCTCTGTGTATTTGTCAA3’ and antisense primer 5′ CCTGGAGGAGAAGAGGAAAGAGA3’ and the enzyme glyceraldehyde 3-phosphate dehydrogenase (GAPDH), sense primer 5’TCGACAGTCAGCCGCATCTTCTTT3’ and antisense primer 5’GCCCAATACGACCAAA TCCG TTGA3’. The housekeeping genes RPL13a and GAPDH were chosen since both genes are expressed in various tissues and cell types and show no significant changes in expression levels between the individual samples used in the present study. The target genes were analyzed using the following primers for HPSE: sense primer 5’TGGCAAGAAGGTCTGGTTAGGAGA3’ and antisense primer 5’GCAAAGGTGTCGGATAGCAAGGG3’; for the amplification of the HPSE-2 isoform, sense primer 5’AGACAGAGCTGCAGGTTTGAAGGA3’ and antisense primer 5’AGCTTAGGAAATCGAGCCAGCCAT3’ and for syndecan-1 sense primer 5’AGGGCTCCTGCACTTACTTGCTTA3’ and reverse primer 5’ATGTGCAGTCATACACTCCAGGCA3’. The values are expressed as (−ΔCt) in triplicate. All of the primers were produced by Applied Biosystems (California, USA). The amplification was carried out using the reagent Maxima® SYBR Green qPCR Master Mix (2X) (Applied Biosystems, California, USA), according to the following modified protocol: 1.5 μM of each primer, 1 μg cDNA, 0.025 μL of the solution ROX 50 μM diluted 10X and 6 μL of SYBR Green 2X. The mixture was transferred to a thermocycler for amplification in real-time (7500 Real-Time PCR Cycler®) (Applied Biosystems, California, USA), with the cycling of 95 °C for 10 min followed by 40 cycles of 95 °C, 15 s; 60 °C, 60 s. The temperature of the melting curve was determined for each pair of primers*.*

### Statistical analysis

Statistical analysis was carried out using GraphPad Prism 5® (GraphPad Prism Software Inc., CA, USA). The values were expressed as means and standard deviation with a significance level of *p* ≤ 0.05. The statistical analyses involved ANOVA and Bonferroni adjustment test to compare different groups and the t-Student test and Dunn’s test for analysis between two groups.

## Results

The demographic characteristics and clinical features (PASI classification) of individuals involved in the present study were shown in Table 1 and Table 2. There are no demographic differences between individuals enrolled in the present study (control and patient groups).

**Table 1:** Demographic Characteristics

Group	Gender	N	%	Age (median ± SD)	*p	**p
Control	male	4	22.0	53.75 ± 14.38	0.5571	
	female	14	78.0	54.64 ± 6.98		
	Total	18	100.0			
						0.9912
Psoriasis	male	11	52.0	42.4 ± 12.0	0.4784	
	female	12	48.0	42.4 ± 12.0		
	Total	23	100.0			

*N* number of samples, *p-value refers to the average age between men and women within each group (control and patients), **p-value refers to the age difference between the control group and the patient group

**Table 2:** Clinical Features

Group	^a^PASI	N	%
Control	no	18	100.0
Psoriasis	mild	10	43.5
	moderate	7	30.5
	severe	6	26.0
	Total	23	100.0

^a^*PASI* Psoriasis Area and Severity Index; PASI < 7 (mild); PASI < 12 (moderate); PASI > 12 (severe)

**Fig. 1:**
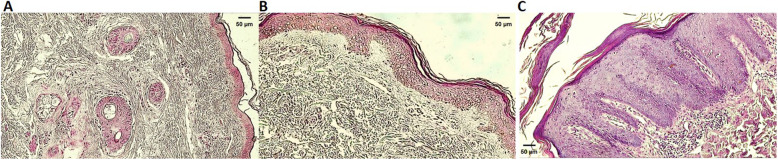
Haematoxylin and Eosin Staining. Differences between skin from individuals not affected by the disease, blepharoplasty tissue **A**, non-affected skin area from patients with psoriasis **B**, and skin from a central area of the psoriasis plaque **C.** Magnified 40X. Micrometric scale 50 μm

We can observe the significant differences between normal skin and skin affected by psoriasis (Fig. 1). Histologically, the epidermis of normal skin is much thinner than with psoriasis (absence of acanthosis). The dermal-epidermal junction is less sinuous than in psoriasis since the dermal papillae and interpapillary crests shorter. The granular layer is present, and there are no nuclei in the corneal layer. The keratinized cells in the section appear reticulated and loose, as in orthokeratosis, in addition to the absence of parakeratosis (Fig. 1A).

In the dermis affected by psoriasis, we frequently find mild to moderate nonspecific chronic inflammatory infiltrate near the vessels, acanthosis, and Munro’s microabscess (Fig. 1B).

**Fig. 2:**
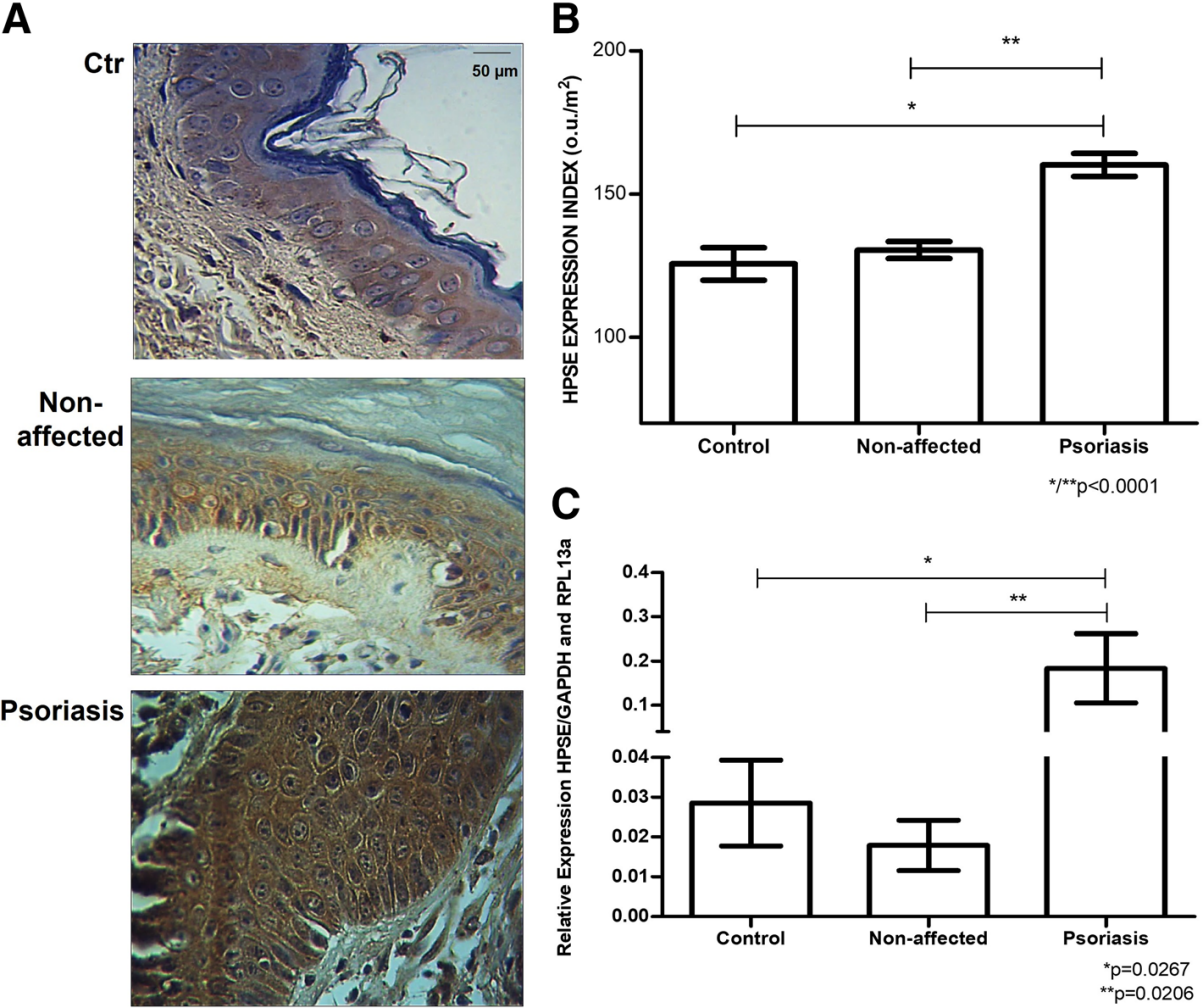
Higher HPSE expression in psoriasis plaque. **A**, Immunostaining using an anti-HPSE antibody (magnified 400X). **B**, Protein expression of HPSE obtained digital quantification (u.o/μm^2^). The values represent mean and standard deviation. **C**, HPSE messenger RNA (mRNA) detection by quantitative RT-PCR. The values represent mean and standard deviation relative to endogenous genes GAPDH (glyceraldehyde-3-fosfate-dehydrogenase) and RPL13a (a ribosomal protein). The analysis was performed in triplicate samples. **Control**, blepharoplasty tissue; **Non-affected**, unaltered skin tissue from a patient with psoriasis; **Psoriasis**, a central area of psoriasis plaque

The labeling of heparanase-1 (HPSE) in the psoriasis plaque was greater than in the control skin 160.16 ± 11.89 ou/μm^2^ versus 125.58 ± 20.56 ou/μm^2^ (*p* < 0.0001), as shown in Fig. 2. There is no difference in HPSE protein expression between Control and Non-affected tissues. However, it is interesting to note that HPSE presents more intense protein staining in the psoriasis plaque than the Non-affected skin area of the patient 160.16 ± 11.89 ou/μm^2^ versus 130.41 ± 10.99 ou/μm^2^ (p < 0.0001), as shown in Fig. 2.

Figure 2 shows that gene expression of the enzyme HPSE was significantly higher in the Psoriasis group than in the Control group 0.183 ± 0.270 versus 0.028 ± 0.031 (*p* = 0.0267). As expected, patients’ Non-affected skin presented lower HPSE mRNA expression than affected skin 0.017 ± 0.016 versus 0.183 ± 0.270 (*p* = 0.0206).

**Fig. 3:**
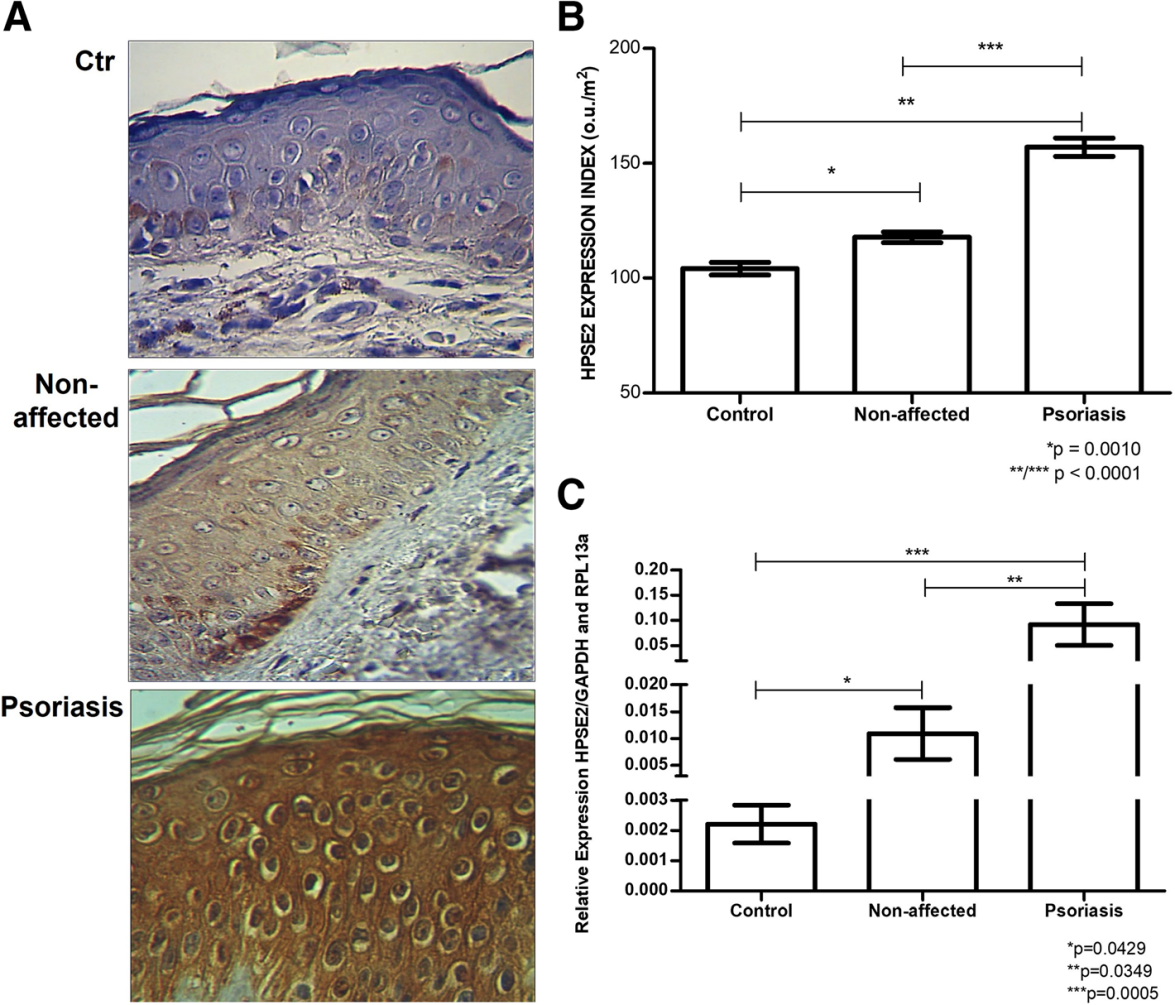
HPSE2 protein level and mRNA expression were increased in skin tissues of patients with psoriasis. **A,** Immunostaining using an anti-HPSE2 antibody (magnified 400X). **B**, HPSE2 protein expression. Mean and standard deviation values were obtained by digital quantification (u.o/μm^2^). **C**, HPSE2 mRNA expression using quantitative RT-PCR. Relative mRNA expression compared to endogenous genes GAPDH (glyceraldehyde-3-fosfate-dehydrogenase) and RPL13a (a ribosomal protein). The mean and standard deviation The values represent the analysis performed in triplicate samples. **Control**, blepharoplasty skin; **Non-affected**, unaltered skin tissue from patient with psoriasis; **Psoriasis**, a central area of psoriasis plaque

It was observed higher protein expression of HPSE2 in the Psoriasis group concerning the Non-affected group 157.06 ± 11.19 ou/μm^2^ versus 117.75 ± 7.49 ou/μm^2^ (*p* < 0.0001) and Psoriasis group compared to Control 157.06 ± 11.19 ou/μm^2^ versus 104.08 ± 9.24 ou/μm^2^ (p < 0.0001), demonstrated in Fig. 3.

We also noted in Fig. 3 a significant difference in the HPSE2 protein expression between Control tissues and Non-affected tissues 104.08 ± 9.24 ou/μm^2^ versus 117.75 ± 7.49 ou/μm^2^ (*p* = 0.0010).

The mRNA expression of HPSE2 was also significantly higher in the Psoriasis group compared to the Non-affected tissue of patients with psoriasis 0.0940 ± 0.1342 versus 0.0109 ± 0.0134 (*p* = 0.0349). We can also observe an increased mRNA expression of HPSE2 in the Psoriasis Group than the Control Group (0.0940 ± 0.1342 versus 0.0023 ± 0.0021) *p* = 0.0005. Interestingly, Non-affected skin from patients with psoriasis has higher HPSE2 expression than the Control Group 0.0109 ± 0.0134 versus 0.0023 ± 0.0021 (*p* = 0.0429), as shown in Fig. 3.

**Fig. 4:**
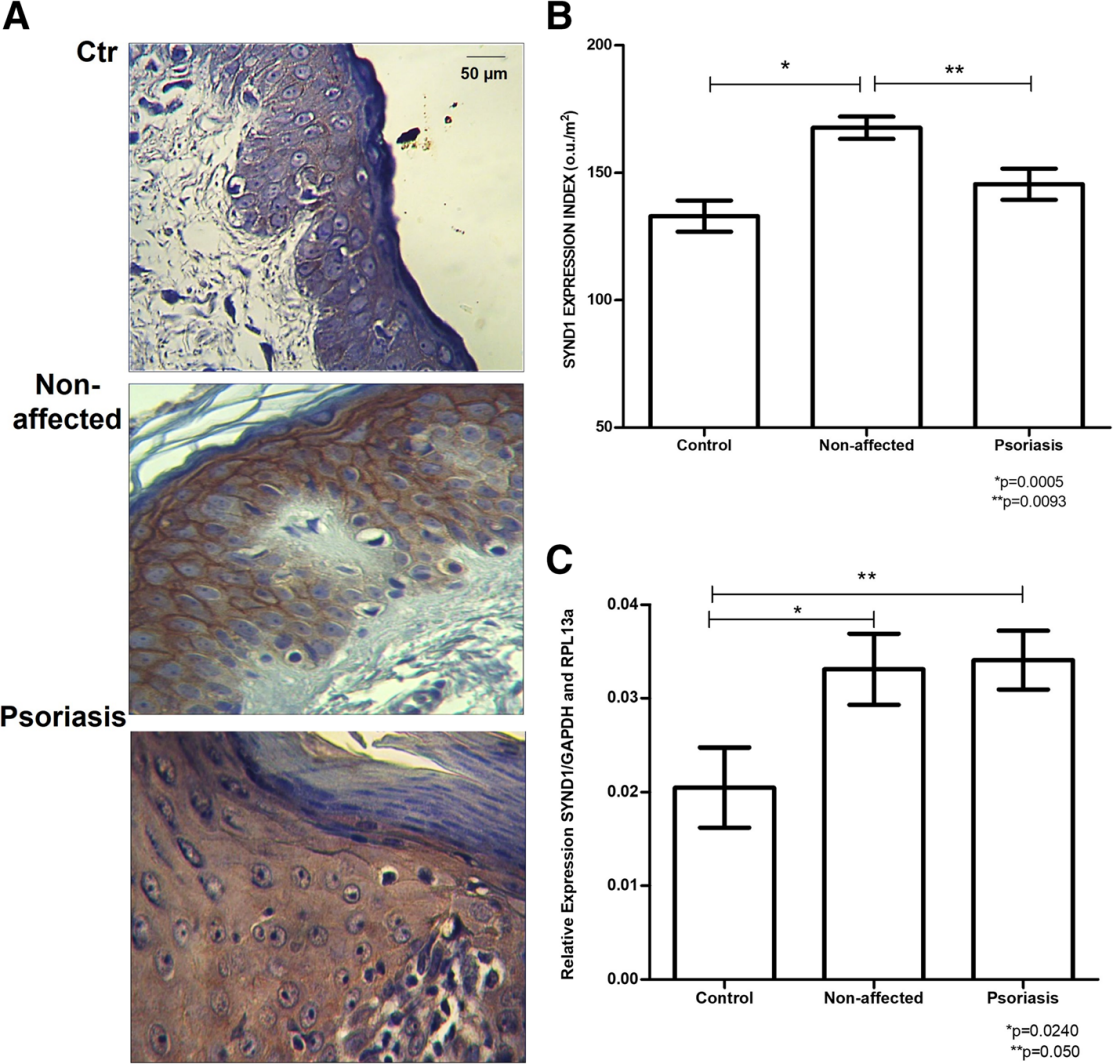
Profile of syndecan-1 (SYND1) in the skin of psoriasis patients compared to control tissue. **A,** Immunostaining using an anti-syndecan-1 antibody (magnified 400X). **B**, SYND1 protein expression. The values represent the mean and standard deviation of protein expression of SYND1 obtained by digital quantification (u.o/μm^2^). **C**, Messenger RNA (mRNA) expression of syndecan-1 obtained by quantitative RT-PCR. The values represent mean and standard deviation relative to endogenous genes GAPDH (glyceraldehyde-3-fosfate-dehydrogenase) and RPL13a (a ribosomal protein). The values represent the analysis performed in triplicate samples. **Control,** skin obtained by blepharoplasty; **Non-affected**, unaltered skin tissue from patient with psoriasis; **Psoriasis**, affected skin from a central area of the psoriasis plaque

The protein expression of the heparan sulfate proteoglycan, syndecan-1, is significantly higher in the non-affected skin samples from patients with psoriasis than skin tissue collected from psoriasis plaque 173.08 ± 7.93 ou/μm^2^ versus 150.34 ± 17.44 ou/μm^2^ (*p* = 0.0093). We can also observe in Fig. 4 an enhance in SYND1 protein expression in Non-affected tissue compared to control samples 173.08 ± 7.93 ou/μm^2^ versus 127.64 ± 14.71 ou/μm^2^ (*p* = 0.0005).

The relative expression of SYND1 mRNA is equally increased in the psoriasis plaque and the Non-affected tissue 0.0341 ± 0.0091 versus 0.0294 ± 0.0126. However, an increase in SYND1 mRNA from tissue not affected by psoriasis was observed compared to the skin samples obtained from blepharoplasty 0.0341 ± 0.0091 versus 0.0204 ± 0.0127 (*p* = 0.0240). A higher level of SYND1 mRNA was also observed in the Psoriasis group than the Control group 0.0294 ± 0.0126 versus 0.0204 ± 0.0127 (*p* = 0.050) shown in Fig. 4C.

**Fig. 5:**
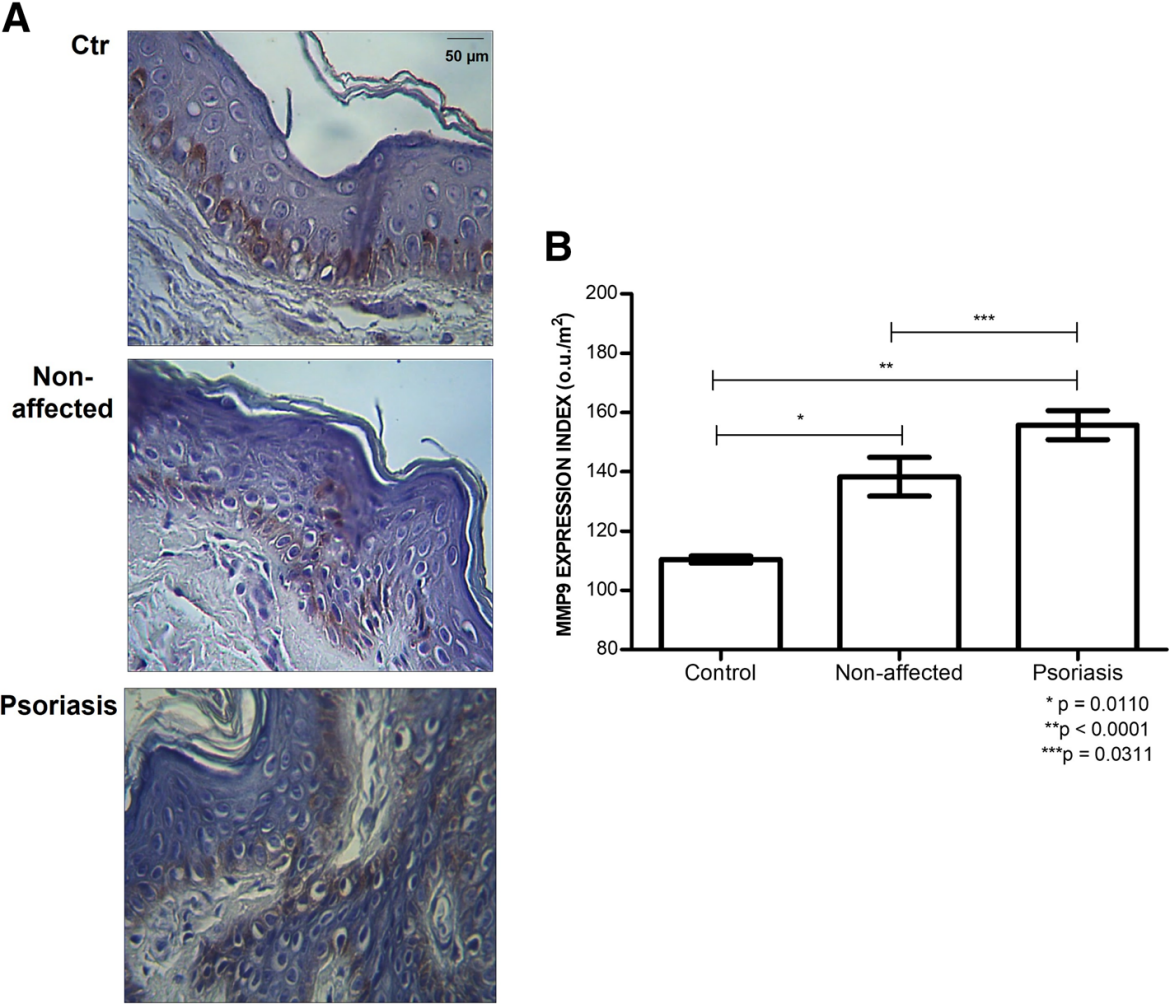
Increased level of protein metalloprotease-9 (MMP9) in psoriasis. **A,** Immunostaining using an anti-MMP9 antibody (magnified 400X). **B**, Protein expression of MMP9 obtained digital quantification (uo/μm^2^). The values indicate the mean and standard deviation of triplicate assays. **Control**, skin obtained by blepharoplasty; **Non-affected**, unaltered skin tissue from patient with psoriasis; **Psoriasis**, skin tissue obtained from psoriasis plaque

An increase in immunolabeling of metalloproteinase-9 (MMP9) in the epidermis of the psoriasis plaque can be seen compared to the Non-affected and Control Groups (Fig. 5A).

Upon quantifying the immunohistochemical reactions, we observed that the protein expression of the enzyme MMP9 was higher in the psoriasis plaque than the skin collected from the Non-affected area 154.69 ± 18.94 ou/μm^2^ versus 136.16 ± 22.92 ou/μm^2^ (*p* = 0.0311). Psoriasis group has also increased protein expression of MMP9 than Control group 154.69 ± 18.94 ou/μm^2^ versus 109.94 ± 1.67 ou/μm^2^ (*p* < 0.0001). It was also shown that non-affected skin obtained from patients with psoriasis presents a higher protein level of MMP9 comparing blepharoplasty tissue 136.16 ± 22.92 ou/μm^2^ versus 109.94 ± 1.6 ou/μm^2^ (*p* = 0.0110), as shown in Fig. 5B.

**Fig. 6:**
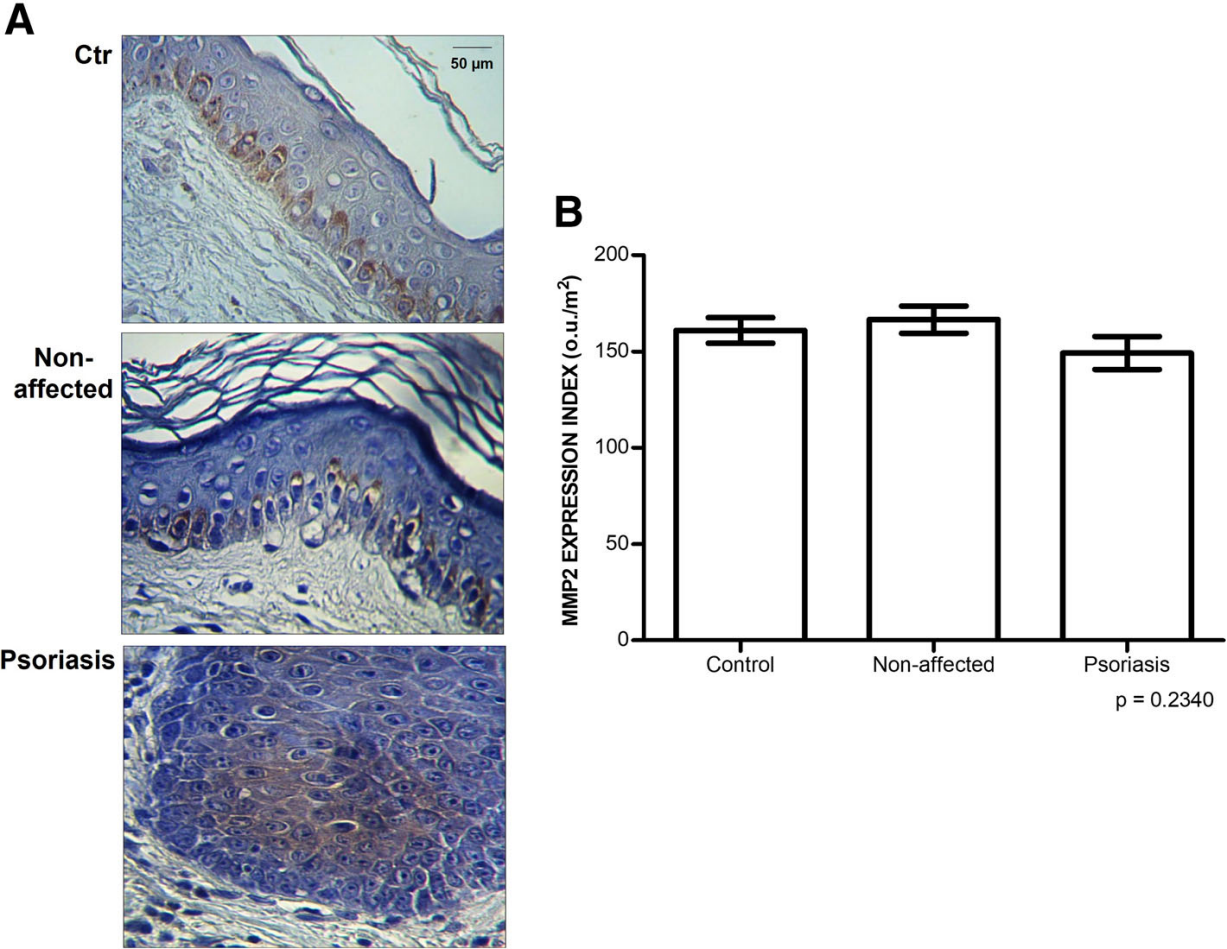
Expression of metalloprotease-2 (MMP2). There was no difference in the MMP2 comparing psoriasis and control skin samples. **A,** Immunostaining using an anti-MMP2 antibody (magnified 400X). B, Protein expression of MMP2 obtained by digital quantification (u.o/μm^2^). The values represent the mean and standard deviation of triplicate assays. Control, blepharoplasty; Non-affected, unaltered skin tissue from patient with psoriasis; Psoriasis, psoriasis plaque sample

The localization of MMP2 in the skin is preferentially in the epidermis, specifically the lowest layer (basal membrane); however, in the skin samples from the Control (blepharoplasty) and the non-affected skin of patients with psoriasis, the immunolabeling is more dispersed throughout the epidermis (Fig. 6A).

There was no difference in the protein expression of MMP2 between the control samples (161.08 ± 16.48) ou/μm^2^, non-affected tissues 166.69 ± 20.39 ou/μm^2^, and psoriasis plaques 149.33 ± 23.22 ou/μm^2^ (Fig. 6B).

The immunolabeling of TIMP2 (Tissue Inhibitor of metalloproteinase-2) is distributed through the extracellular matrix of the epidermis, apparently with a similar intensity of expression between the control, non-affected, and affected samples, although the highest intensity staining was found in the samples obtained from patients.

**Fig. 7:**
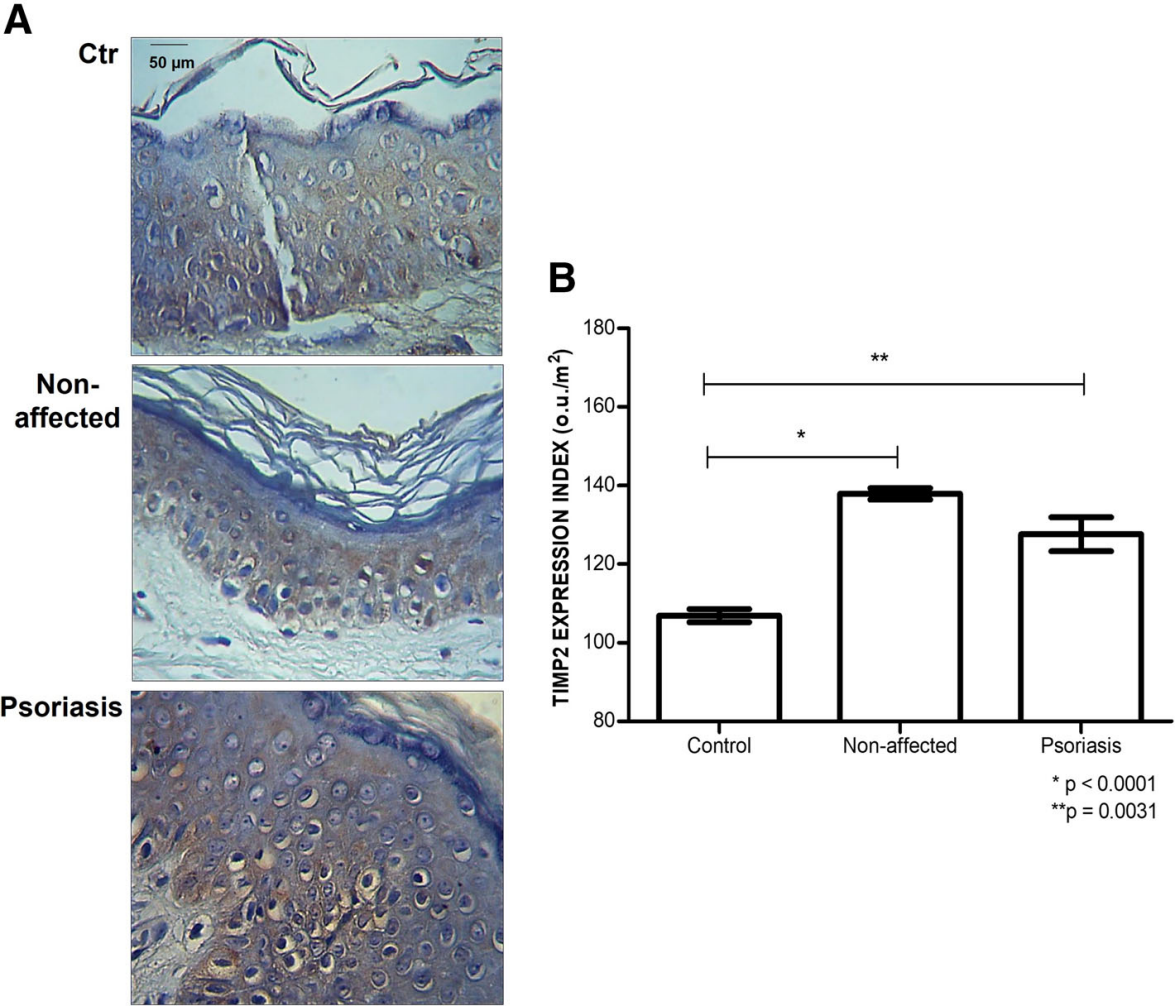
Higher level of TIMP2 (*Tissue inhibitor 2 of metalloprotease*) in psoriasis. **A,** Immunostaining using an anti-TIMP2 antibody (magnified 400X). **B**, protein expression of TIMP2 obtained by digital quantification (uo/μm^2^). The values indicate the mean and standard deviation of triplicate assays. **Contro**l, blepharoplasty skin; **Non-affected**, unaltered skin area from patient with psoriasis; **Psoriasis**, skin tissue obtained from psoriasis plaque

After the quantitative digital analysis, the protein expression of TIMP2 was found to be higher in the samples of not affected skin from psoriasis patients than in Control tissues 137.16 ± 4.45 ou/μm^2^ versus 107.16 ± 4.18 ou/μm^2^ (*p* < 0.0001). We can also verify in Fig. 7B an increase in protein expression of TIMP2 in the Psoriasis group with samples from the Control group 127.57 ± 15.59 ou/μm^2^ versus 107.16 ± 4.18 ou/μm^2^ (*p* = 0.0031).

We did not observe any significant change in the protein and mRNA expression of all studied molecules in the group of patients with different PASI classifications (mild, moderate, and severe (Supplementary Table 1 and Supplementary Table 2).

## Discussion

Psoriasis lesions are characterized by hyper-proliferation of epidermal cells such as keratinocytes and melanocytes associated with inflammatory cellular infiltrate in the dermis and epidermis.

The effect of HPSE on the promotion of angiogenesis [27], the elevation of vascular permeability [28], extravasation of leukocytes [29, 33], and release of cytokines associated with heparan sulfate oligosaccharides generated by the enzymatic activity of HPSE [34] is well known. HPSE also participates in the activation of macrophages [35]. Such events are associated with inflammation, indicating that the enzyme HPSE plays a crucial role in the inflammatory process.

It has been reported that HPSE is preferentially expressed in human psoriatic lesions [30]. Furthermore, over-expression of HPSE promotes mouse skin lesions as that strongly recapitulate human psoriasis in terms of histomorphological appearance and cellular characteristics [30]. The fact that an inflammatory process characterizes psoriasis, as demonstrated by the significant increase in the gene and protein expression of HPSE in the psoriatic plaque, suggest that HPSE is possibly involved in the triggering and maintenance of the inflammatory state characteristic of psoriasis, therefore presenting a potential target for therapy as already proposed for other diseases such as cancer [36, 37].

Clinical tests directed at HPSE in malignant diseases are under intense development [38, 39] and could become highly beneficial to patients with psoriasis. Other studies designed to gain a detailed understanding of the possible molecular and cellular events modulated by HPSE that occur in the skin affected by psoriasis, together with a systematic analysis of this enzyme’s inhibitors, may be key to developing effective therapeutic strategies for psoriasis.

Although there are no reports in the literature that correlates psoriasis with HPSE2, it is known that HPSE2 shows no catalytic activity over heparan sulfate but presents a high affinity for heparin and heparan sulfate, which can modulate the activity of the enzyme HPSE [40].

Our study was a pioneer in showing higher levels of HPSE2 expression in the patient’s skin area affected by psoriasis plaque. There was also increased expression of HPSE2 even in a non-psoriatic area from patients with psoriasis, showing that HPSE2 might be highly expressed in the initial stages of the disease.

Heparan sulfate proteoglycan syndecan-1 (SYND1) plays a critical role in regulating psoriasis-like skin inflammation in mice [26]. It has been demonstrated in the literature that the release of heparan sulfate proteoglycan syndecan-1 from the cell surface depends on the action of MMP9 together with the action of HPSE and, more importantly, that HPSE activity can lead to increased levels of proteases [41].

Taken together, the significant changes in the expression of HPSE, HPSE2, SYND1 in the skin affected by psoriasis confirm alterations of ECM in such disease.

It is known that tissue remodeling requires MMPs. These proteases are associated with increased permeability of blood capillaries and loss of cell-cell interaction, essential characteristics for psoriasis development [26, 42].

Psoriasis shares a systemic production of inflammatory mediators, which are key regulators of the production of matrix metalloproteinase (MMP9) and its inhibitor TIMP1 [43]. It has been described in the literature that MMPs, including MMP9 levels, are higher in the serum of patients with psoriasis. The increase of MMP9 in psoriasis patients’ blood-circulating cells is another piece of evidence that this disease presents systemic characteristics [43]. MMP9 is regulated by tumor necrosis factor-alpha (TNF-α), which plays a central role in psoriasis [44].

Interestingly, Lindqvist and coworkers found an increase and displacement of MMP9 when comparing normal skin from healthy individuals and psoriatic skin of a methotrexate-treated patient with polyarthritic psoriatic arthritis [45].

The increase in protein levels of MMP9 in the tissue affected by psoriasis, observed in the present study, corroborates the results described in the literature. However, it is important to note that the protein expression of MMP9 is also significantly higher in the non-affected tissue of patients with psoriasis compared to the control group. It is a novel result in the literature, suggesting that the change in expression of MMP9 is a systemic event and not just a feature of the affected region, indicating that this protease could serve as a marker for the disease even when the patient does not present the active disease, that is, does not present psoriasis plaques on the skin.

As cited in the literature, high levels of MMP9 may also cause delays in the re-epithelialization of skin [46]. Presumably, high levels of MMP9 in the psoriasis plaque lead to the rupture of the extracellular matrix, hindering its remodeling and, consequently, maintaining an inflammatory microenvironment for the rapid and inappropriate replication of keratinocytes.

A great effort has been made to state biochemical biomarkers for the diagnosis of psoriasis. A set of biomarkers, composed matrix metalloproteinases, and their tissue inhibitors comprises MMP2, MMP12, MMP13, TIMP2, and TIMP4 whose circulating levels are higher psoriasis undergoing systemic therapy compared to the parallel with psoriatic arthritis cohort [47].

Studies show that the overexpression of MMP2 in the psoriatic epidermis remains contradictory. Fleischmajer and collaborators showed an increase in the expression of MMP2 and TIMP2 in the skin of psoriasis patients [48], while other researchers showed an increase in the plasma levels of MMP2 and TIMP2 in psoriasis patients compared to the level observed in the plasma of healthy individuals [49]. These results represent strong evidence that the high concentration of MMP2, without a commensurate increase in its tissue inhibitor (TIMP2), can lead to neoangiogenesis and, consequently, the dissemination of psoriatic lesions. The high concentration of MMP2 during psoriasis eruption and its significant reduction after successful treatment and eliminating symptoms of the disease indicate that this enzyme may be associated with psoriasis [49].

TIMPs 1, 2, 3, and 4 form a group of tissue inhibitors of metalloproteinases. These proteins’ main function is to inhibit the adverse effects caused by the overexpression of MMPs [50]. It has been verified that, under physiological conditions, the MMP/TIMP ratio is highly regulated. An imbalance in the MMP/TIMP value can result in pathological production and cleaving of the extracellular matrix and basal membrane [51].

Our results showed an increase in the expression of TIMP2 in psoriasis patients, both in affected and non-affected skin, compared to control samples (blepharoplasty). This result is in agreement with that described by other authors [48].

The genes for MMP2 and TIMP2 are constitutively expressed in many cells during embryogenesis and adult life [52, 53]. Changes in cell-cell and cellular matrix adhesion in the psoriatic epidermis, associated with an increase in the MMP2-TIMP2 complex, suggest that these compounds can regulate the proteolysis of keratinocyte adhesion molecules and play a role in their transepidermal migration [48].

It was described that TIMP2 is directly involved in the activation of MMP2 [53–55]. The main activation of proMMP-2 occurs on the cell surface and is mediated by Membrane Type-Metalloproteinases (MT-MMPs), including MT1-MMP, and such activation depends on the assistance of TIMP-2. ProMMP-2 forms a tight complex with TIMP-2 and MT1-MMP on the cell surface. An MT1-MMP then activates the cell surface-bound proMMP-2. The ternary complex effectively promotes MMP-2 activation [55].

It is important to highlight that the anti-MMP2 antibody used in our assay does not differentiate between the inactive and active forms of MMP2. The fact that higher MMP2 expression was not observed in the psoriatic tissue than the control tissue does not exclude the possibility of a higher proportion of active MMP2 in psoriasis tissue that might occur due to increased expression of TIMP2, as already reported in the literature [48, 55].

The activation of MMP2 promotes remodeling of the extracellular matrix in inflammatory processes, characteristic of the psoriasis condition. TIMP2 may be a key molecule in the activation of MMP2 in psoriasis and, in this manner, contribute to the inflammatory process.

## Conclusions

The significant increase in the expression of heparanase-1 (HPSE), heparanase-2 (HPSE2), syndecan-1 (SYND1), metalloproteinase-9 (MMP9), and tissue inhibitor of metalloproteinase 2 (TIMP2) in biopsies of skin affected by psoriasis showed extracellular matrix alterations in psoriasis.

HPSE2, SYND1, MMP9, and TIMP2 have higher protein expression in the non-affected skin tissue of patients with psoriasis than control tissues, indicating that increased expression of such molecules possibly occurs in the initial stages of the disease even in the absence of psoriatic plaque.

The data shows significant changes in the extracellular matrix in the sample tissues collected from patients affected by psoriasis and opens the possibility of therapeutic approaches directed at these molecules.

### Limitation of the study

Gene expression may vary in different parts of the body, so it would be better if samples of skin from healthy individuals could be obtained from the same site involved in patients with psoriasis. However, as it may characterize an unnecessary procedure for such individuals, we decided to collected tissue from blepharoplasty as control.

## Supplementary Information

**Additional file 1.**

**Additional file 2.**

## Data Availability

The datasets used and/or analyzed during the current study are available from the corresponding author on reasonable request.

## Abbreviations

HPSE: Heparinase-1
HPSE2: Heparinase-2
SYND1: Syndecan-1
MMP9: Metalloproteinase-9
MMP2: Metalloproteinase-2
TIMP2: Tissue inhibitor of metalloproteinase 2
PASI: Psoriasis Area and Severity Index
