# Supplementary figures and images for: Extracellular matrix alterations in the skin of patients affected by psoriasis
Source: BMC Mol Cell Biol. 2021 Oct 29;22:55. doi: 10.1186/s12860-021-00395-1 (PMC8555298; doi:10.1186/s12860-021-00395-1)

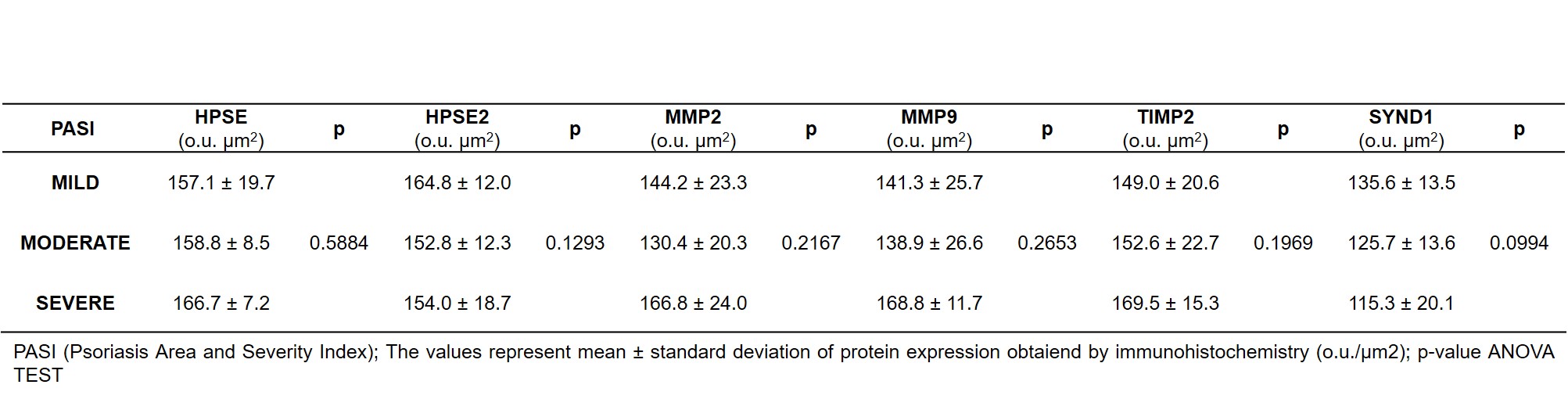

Supplement: Supplementary file 1 — Additional file 1. [file 12860_2021_395_MOESM1_ESM.jpg]

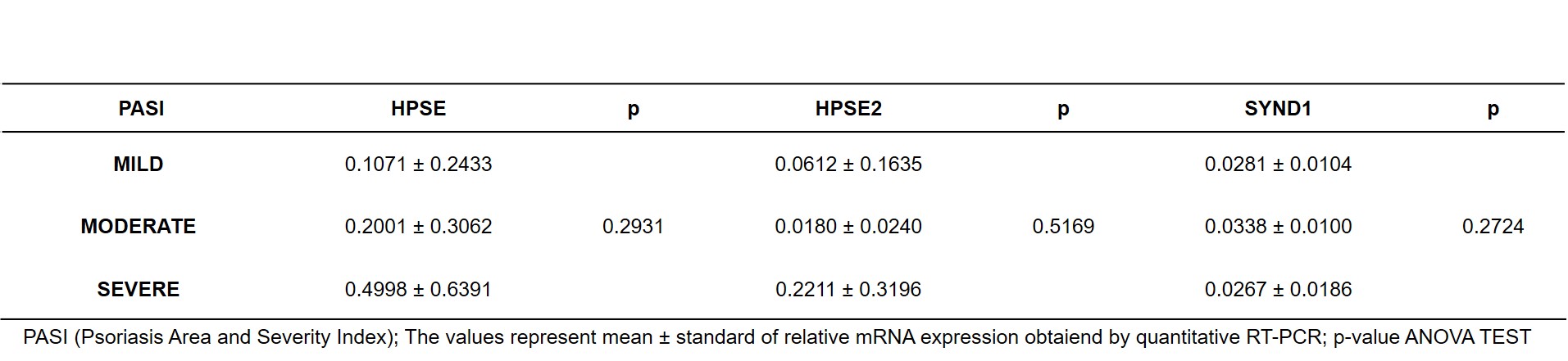

Supplement: Supplementary file 2 — Additional file 2. [file 12860_2021_395_MOESM2_ESM.jpg]
